# High LGALS3 expression induced by HCP5/hsa-miR-27b-3p correlates with poor prognosis and tumor immune infiltration in hepatocellular carcinoma

**DOI:** 10.1186/s12935-024-03309-1

**Published:** 2024-04-20

**Authors:** Yinghui Ren, Yongmei Qian, Qicheng Zhang, Xiaoping Li, Mingjiang Li, Wei Li, Pan Yang, Hengchang Ren, Hongxia Li, Yiqi Weng, Dengwen Li, Ke Xu, Wenli Yu

**Affiliations:** 1 Department of Anesthesiology, Tianjin First Central Hospital, Tianjin, 300192 China; 2 Tianjin Key Laboratory of Lung Cancer Metastasis and Tumor Microenvironment, Tianjin Lung Cancer Institute, Tianjin Medical University General Hospital, Tianjin, 300052 China; 3 Department of Thoracic Surgery, Tianjin First Central Hospital, Tianjin, 300192 China; 4 College of Life Sciences, Nankai University, Tianjin, 300071 China

**Keywords:** LGALS3, Hepatocellular carcinoma, HCP5, Immune cell infiltration, Prognosis, Tumor microenvironment

## Abstract

**Background:**

Hepatocellular carcinoma (HCC) is widely recognized for its unfavorable prognosis. Increasing evidence has revealed that LGALS3 has an essential function in initiating and developing several malignancies in humans. Nevertheless, thorough analysis of the expression profile, clinical prognosis, pathway prediction, and immune infiltration of LGALS3 has not been fully explored in HCC.

**Methods:**

In this study, an initial pan-cancer analysis was conducted to investigate the expression and prognosis of LGALS3. Following a comprehensive analysis, which included expression analysis and correlation analysis, noncoding RNAs that contribute to the overexpression of LGALS3 were subsequently identified. This identification was further validated using HCC clinical tissue samples. TIMER2 and GEPIA2 were employed to examine the correlation between LGALS3 and HCP5 with immunological checkpoints, cell chemotaxis, and immune infiltration in HCC. The R program was applied to analyze the expression distribution of immune score in in HCC patients with high and low LGALS3 expression. The expression profiles of immune checkpoints were also analyzed. Use R to perform GSVA analysis in order to explore potential signaling pathways.

**Results:**

First, we conducted pan-cancer analysis for LGALS3 expression level through an in-depth analysis of public databases and found that HCC has a high LGALS3 gene and protein expression level, which were then verified in clinical HCC specimens. Meanwhile, high LGALS3 gene expression is related to malignant progression and poor prognosis of HCC. Univariate and multivariate analyses confirmed that LGALS3 could serve as an independent prognostic marker for HCC. Next, by combining comprehensive analysis and validation on HCC clinical tissue samples, we hypothesize that the HCP5/hsa-miR-27b-3p axis could serve as the most promising LGALS3 regulation mechanism in HCC. KEGG and GO analyses highlighted that the LGALS3-related genes were involved in tumor immunity. Furthermore, we detected a significant positive association between LGALS3 and HCP5 with immunological checkpoints, cell chemotaxis, and immune infiltration. In addition, high LGALS3 expression groups had significantly higher immune cell scores and immune checkpoint expression levels. Finally, GSVA analysis was performed to predict potential signaling pathways linked to LGALS3 and HCP5 in immune evasion and metabolic reprogramming of HCC.

**Conclusions:**

Our findings indicated that the upregulation of LGALS3 via the HCP5/hsa-miR-27b-3p axis is associated with unfavorable prognosis and increased tumor immune infiltration in HCC.

**Supplementary Information:**

The online version contains supplementary material available at 10.1186/s12935-024-03309-1.

## Introduction

Hepatocellular carcinoma (HCC) represents the greatest widespread kind of primary liver malignancy (75–85%) and the third most prominent malignancy-caused mortality worldwide [1]. It is known that chronic inflammation or hepatocellular injury is associated with HCC [2]. So far, surgery, chemotherapy, and radiotherapy are the three main therapeutic methods for HCC [3]. In the age of precision medicine, personalized immunotherapy offers hope to patients with limited treatment options in a tumor-agnostic approach [4, 5]. Despite significant progress in immunotherapy in the last few years, many patients remain unable to benefit from immunotherapy, perhaps due to the immunosuppressive mechanisms in the tumor microenvironment (TME) [6]. Tumor cells actively participate in intercellular communication with diverse components of the TME, thereby facilitating tumor progression [7]. Therefore, urgent research into promising prognostic biomarkers and the molecular pathway lying beneath the malignant progression of HCC is crucial for developing successful cancer treatments.

LGALS3 (galectin-3), a galactose-specific lectin, participates in the transport mechanism of integrins, receptors, and other molecules crucial to cell adhesion [8]. LGALS3 also plays a key role in TME immunosuppression and modulates a diverse range of cellular functions, involving cancer biology and cellular homeostasis [9]. Previous studies suggested that LGALS3 abnormal expression throughout cancer progression promotes tumor growth, invasiveness, metastasis, as well as immune suppression. This makes LGALS3 a promising therapeutic target to regulate anti-tumor immunity in several kinds of cancer, including glioma, breast, lung, as well as prostate tumors [9, 10]. It has been well documented that LGALS3 could be a biomarker for prognosis and treatment targets as well as a novel molecule responsible for HCC progression [11–13]. Zhang et al. [14] suggested overexpression of LGALS3 promoted HCC bone metastasis and induced associated skeletal complications. Nevertheless, the expression, prognosis, epigenetic, and molecular regulatory mechanisms of LGALS3 in HCC have been incompletely studied. In addition, LGALS3 relation with immune infiltration in HCC TME has yet to be inadequately investigated.

This work began with a pan-cancer study of LGALS3 expression and its predictive value in a variety of human malignancies. We further explored the LGALS3 potential upstream regulatory noncoding RNAs (ncRNAs) involving microRNAs (miRNAs) as well as long noncoding RNAs (lncRNAs) throughout HCC. Subsequently, in HCC, a correlation analysis was investigated between LGALS3 and tumor immunity-related indicators involving cell chemotaxis, immune checkpoints, immune cell biomarkers, and infiltration. Eventually, the association between the expression of LGALS3 and signaling pathways was examined in HCC. Findings demonstrated that LGALS3 might have a role in the malignancy of HCC and immune cell infiltration via the HCP5/hsa-miR-27b-3p/LGALS3 axis, suggesting that a novel HCP5/hsa-miR-27b-3p/LGALS3 axis could be a biomarker for prognosis and treatment target for HCC patients.

## Results

### Pan-cancer analysis of LGALS3 expression

**Fig. 1:**
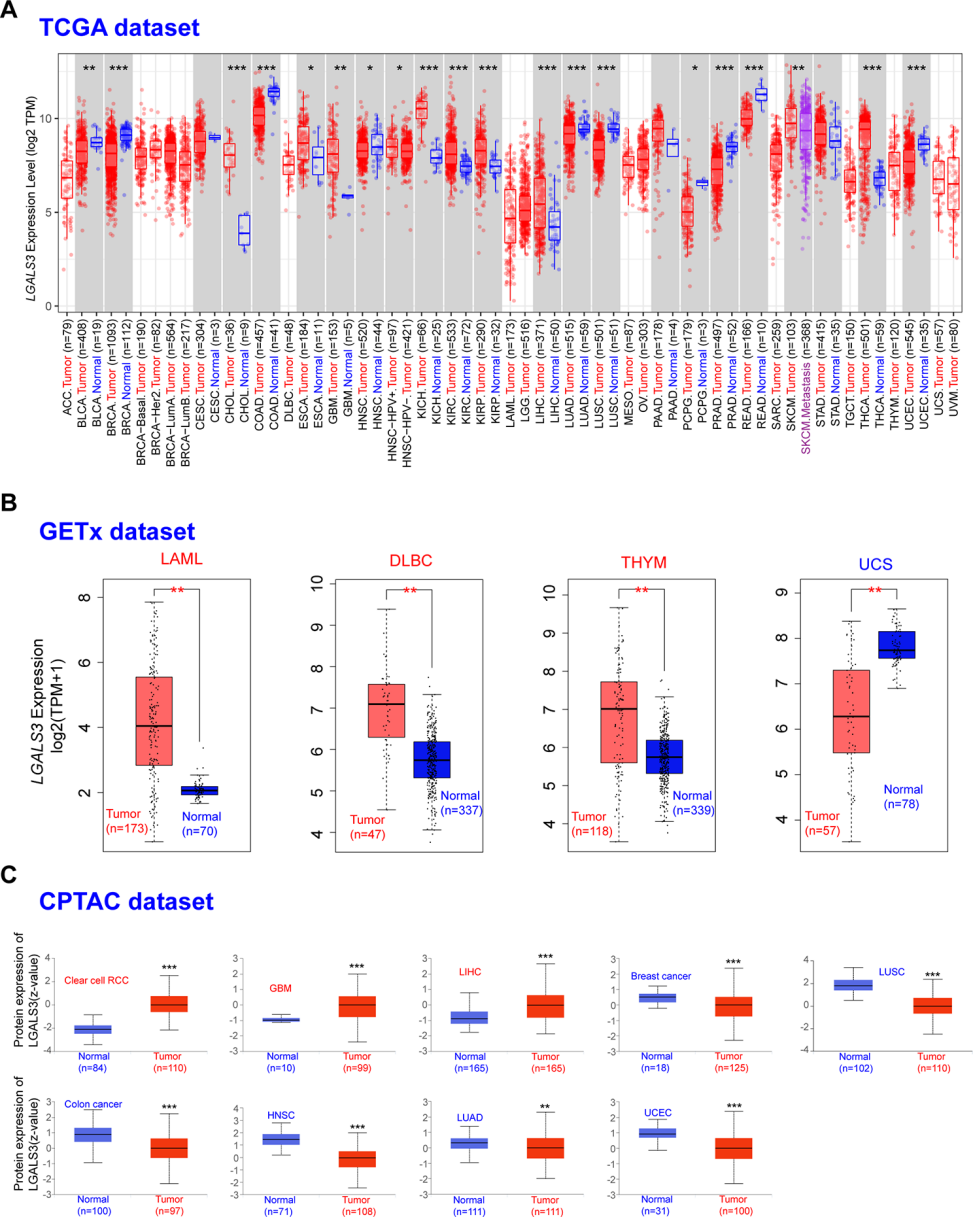
Expression level of LGALS3 gene in different tumors. (**A**) The expression of the LGALS3 gene in different cancers or specific cancer subtypes was assessed by TIMER2 (data from TCGA). (**B**) For the type of LAML, DLBC, THYM, and UCS in the TCGA database, the normal control samples of the GTEx database were included as controls. (**C**) The expression level of LGALS3 protein between normal tissue and primary tissue of clear cell RCC, GBM, LIHC, breast cancer, colon cancer, HNSC, LUAD, LUSC, and UCEC analyzed by CPTAC dataset. *p value < 0.05; **p value < 0.01; ***p value < 0.001

For the investigation of LGALS3’s potential functions in carcinogenesis, TIMER2 was utilized to explore LGALS3 expression among different cancer types of TCGA (The Cancer Genome Atlas). LGALS3 was significantly overexpressed in ten types of cancer compared to matched control tissues, as displayed in Fig. 1A, involving CHOL, KICH, GBM, KIRC, ESCA, KIRP, SKCM, THCA, UCEC, and LIHC, and was significantly decreased in nine cancer forms, involving BRCA, BLCA, COAD, LUAD, HNSC, LUSC, PCPG, PRAD, and READ. Other tumors, such as CESC, PAAD, as well as STAD, were unable to show a significant difference. After utilizing Genotype-Tissue Expression (GTEx), a normal tissue expression dataset, we investigated the expression variation of LGALS3 across normal and malignant tissues of DLBC, LAML, THYM, and UCS (Fig. 1B). However, no significant difference of LGALS3 in ACC, LGG, OV, SARC, or TGCT was observed (Figure S1). Overall, LGALS3 expression was increased within CHOL, GBM, ESCA, KICH, KIRP, KIRC, LIHC, SKCM, THCA, UCEC, DLBC, LAML, and THYM and decreased within BRCA, BLCA, HNSC, COAD, LUSC, LUAD, PCPG, PRAD, READ, and UCS, indicating that LGALS3 may have crucial functions in the carcinogenesis and progression of these 23 cancer types. According to the available Clinical Proteomic Tumor Analysis Consortium (CPTAC) dataset, we determined the expression levels of LGALS3 protein in nine of the 23 cancer types. LGALS3 total protein expression was found to be higher in clear cell RCC primary tissues, GBM, and LIHC, and lesser in the breast cancer, colon cancer, LUAD, HNSC, LUSC as well as UCEC primary tissues than in normal tissues (Fig. 1C).

### LGALS3 prognostic values in human malignancy

**Fig. 2:**
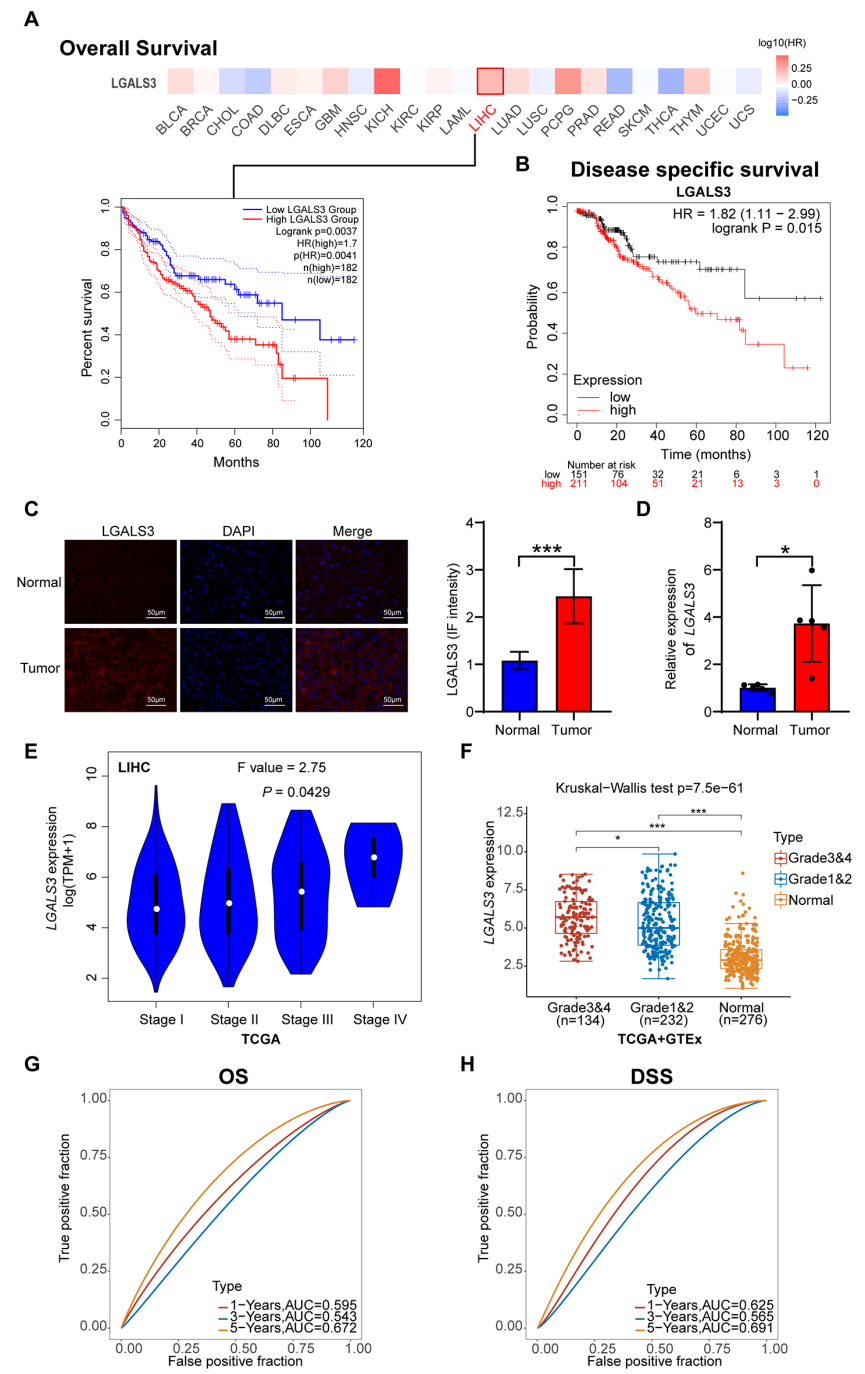
Correlation between LGALS3 gene expression and survival prognosis of various human cancers. (**A**) We used GEPIA2 to obtain the OS analyses of LGALS3 across the 23 types of cancer. The survival map and Kaplan-Meier curves with positive results are offered. (**B**) The prognostic value of LGALS3 in HCC was assessed according to DSS by Kaplan–Meier plotter. (**C**) Representative immunofluorescence images of LGALS3 protein in HCC tissues and adjacent normal tissues. Scale bar, 50 μm. Immunofluorescence image quantification results (right). (**D**) qRT-PCR analysis of LGALS3 expression in tumor and adjacent normal tissues from 5 HCC patients. (**E**) The differential expression of LGALS3 in HCC with main pathological stages (stage I, stage II, stage III, and stage IV) assessed by GEPIA2. (**F**) The expression distribution of LGALS3 in normal tissues and HCC with various tumor grades. (**G**) ROC curve for LGALS3 in predicting 1-, 3-, and 5-year OS. (**H**) ROC curve for LGALS3 in predicting 1-, 3-, and 5-year DSS. The higher values of AUC corresponding to higher predictive power. *p value < 0.05; ***p value < 0.001

Next, the prognostic value of LGALS3 expression in the 23 kinds of cancer patients was then determined. Correlations between LGALS3 expression with OS (overall survival) were evaluated using the GEPIA2 database. In the OS study, only elevated LGALS3 expression indicated poorer survival for HCC patients (Fig. 2A). LGALS3 was not statistically significant for OS of 22 other cancer types patients. Furthermore, DSS (disease-specific survival) was lesser in patients suffering from HCC having higher levels of LGALS3 expression (Fig. 2B). Next, we validated the expression levels of LGALS3 protein in HCC tissues using IF staining. As expected, HCC tumor demonstrated strong LGALS3 expression (Fig. 2C). These findings were further validated by qRT–PCR assay of tumor and adjacent normal tissues from 5 HCC patients. Here, LGALS3 expression was also significantly increased in the HCC tissues (Fig. 2D). In addition, LGALS3 expression was shown to be linked with the pathological stage of HCC, as illustrated in Fig. 2E. High expression of LGALS3 gene is associated with high tumor grade in HCC (Fig. 2F). Moreover, LGALS3 expression was significantly associated with OS and DSS in both univariate and multivariate analyses (Figure S2A-H). Time-dependent ROC analysis showed that the area under the ROC curve was 0.672 at 5 years of OS, and 0.691 at 5 years of DSS (Fig. 2G–H). Taken together, LGALS3 might function as a prospective biomarker for the prognosis of patients suffering from HCC.

### Prediction and analysis of upstream miRNAs of LGALS3

**Fig. 3:**
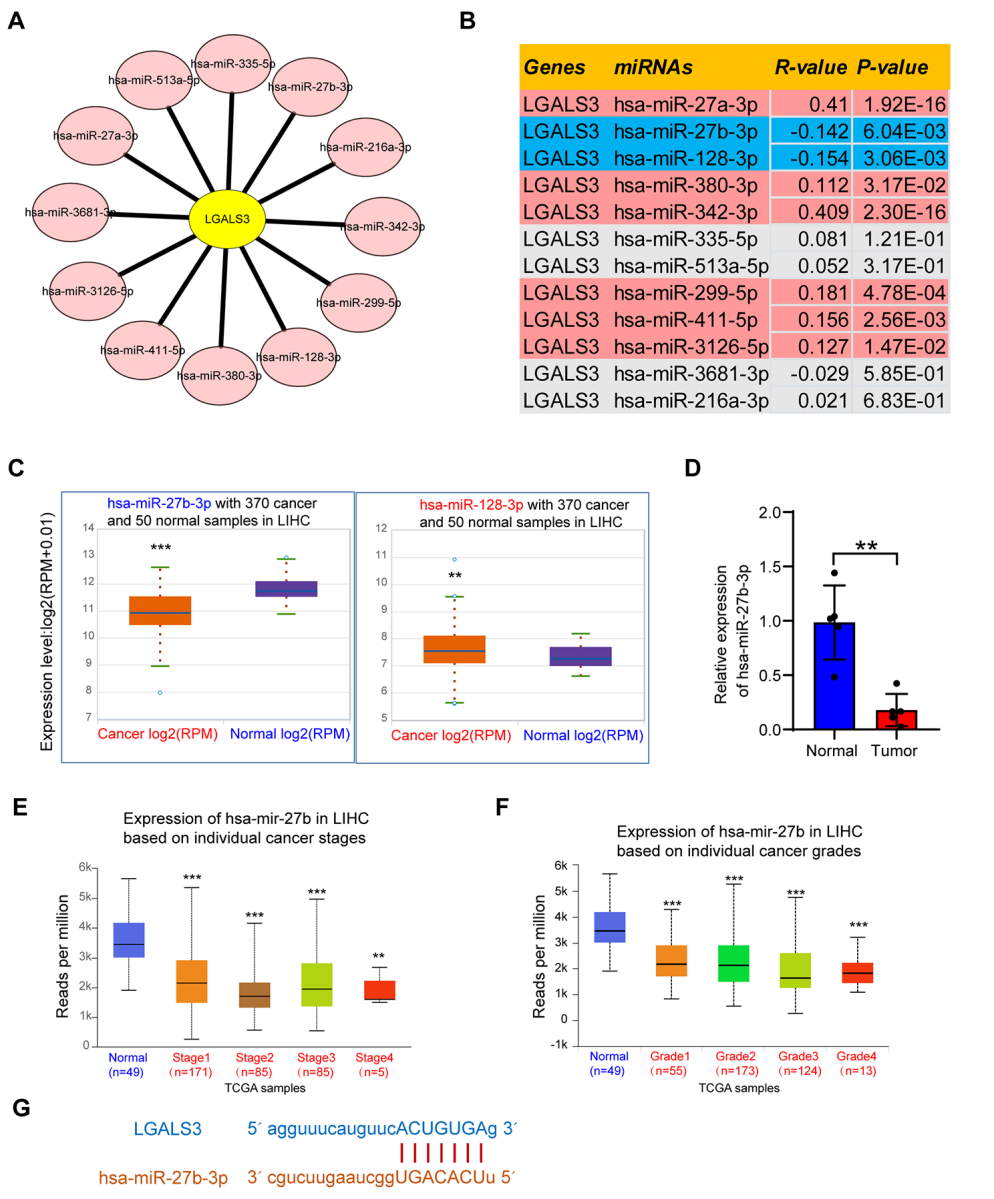
Identification of hsa-miR-27b-3p as a potential upstream miRNA of LGALS3 in HCC. (**A**) Candidate miRNAs interacting with LGALS3 mRNA established by Cytoscape software. (**B**) Correlation analysis between LGALS3 expression and predicted miRNAs in HCC based on the ENCORI database. (**C**) The expression of hsa-miR-27b-3p and hsa-miR-128-3p in HCC and corresponding normal tissues analyzed by ENCORI database. (**D**) qRT-PCR analysis of hsa-miR-27b-3p expression in tumor and adjacent normal tissues from 5 HCC patients. The differential expression of hsa-miR-27b in HCC with various tumor stages (**E**) and tumor grades (**F**) assessed by UALCAN. (**G**) Schematic representation of the hsa-miR-27b-3p target sequence within the 3′UTR of LGALS3. **p value < 0.01; ***p value < 0.001

Throughout the transcriptional and post-transcriptional levels, various ncRNAs have been shown to play a crucial role in regulating gene expression [15]. To assess whether LGALS3 was altered by ncRNAs, we forecasted the possible upstream miRNAs of LGALS3 and subsequently identified 12 miRNAs. Cytoscape software was employed to construct a miRNA-LGALS3 regulatory network in order to enhance visualization (Fig. 3A). MiRNAs are supposed to be negatively correlated with LGALS3 according to their action mechanism. Figure 3B presents that the expression correlation analysis revealed that LGALS3 adversely associated with hsa-miR-27b-3p and hsa-miR-128-3p and positively correlated with hsa-miR-27a-3p, hsa-miR-380-3p, hsa-miR-342-3p, hsa-miR-299-5p, hsa-miR-411-5p, and hsa-miR-3126-5p throughout HCC. There were no statistically significant associations between LGALS3 and the remaining four anticipated miRNAs expression. Further determination of these two miRNA expression levels revealed that only hsa-miR-27b-3p was significantly underexpressed in HCC (Fig. 3C). Subsequently, this finding was validated by qRT–PCR assay of tumor and adjacent normal tissues from 5 HCC patients. Here, hsa-miR-27b-3p expression was also significantly underexpressed in HCC (Fig. 3D). Finally, the hsa-miR-27b-3p expression in various stages and grades of HCC was then identified. Hsa-miR-27b-3p was significantly underexpressed in various pathological stages and grades of HCC (Fig. 3E–F). In addition, Fig. 3G displays the expected interaction among hsa-miR-27b-3p and LGALS3. The aforementioned outcomes revealed that hsa-miR-27b-3p could regulate the expression of LGALS3 and contribute to the progression of HCC.

### Prediction and analysis of upstream lncRNAs of hsa-miR-27b-3p

**Fig. 4:**
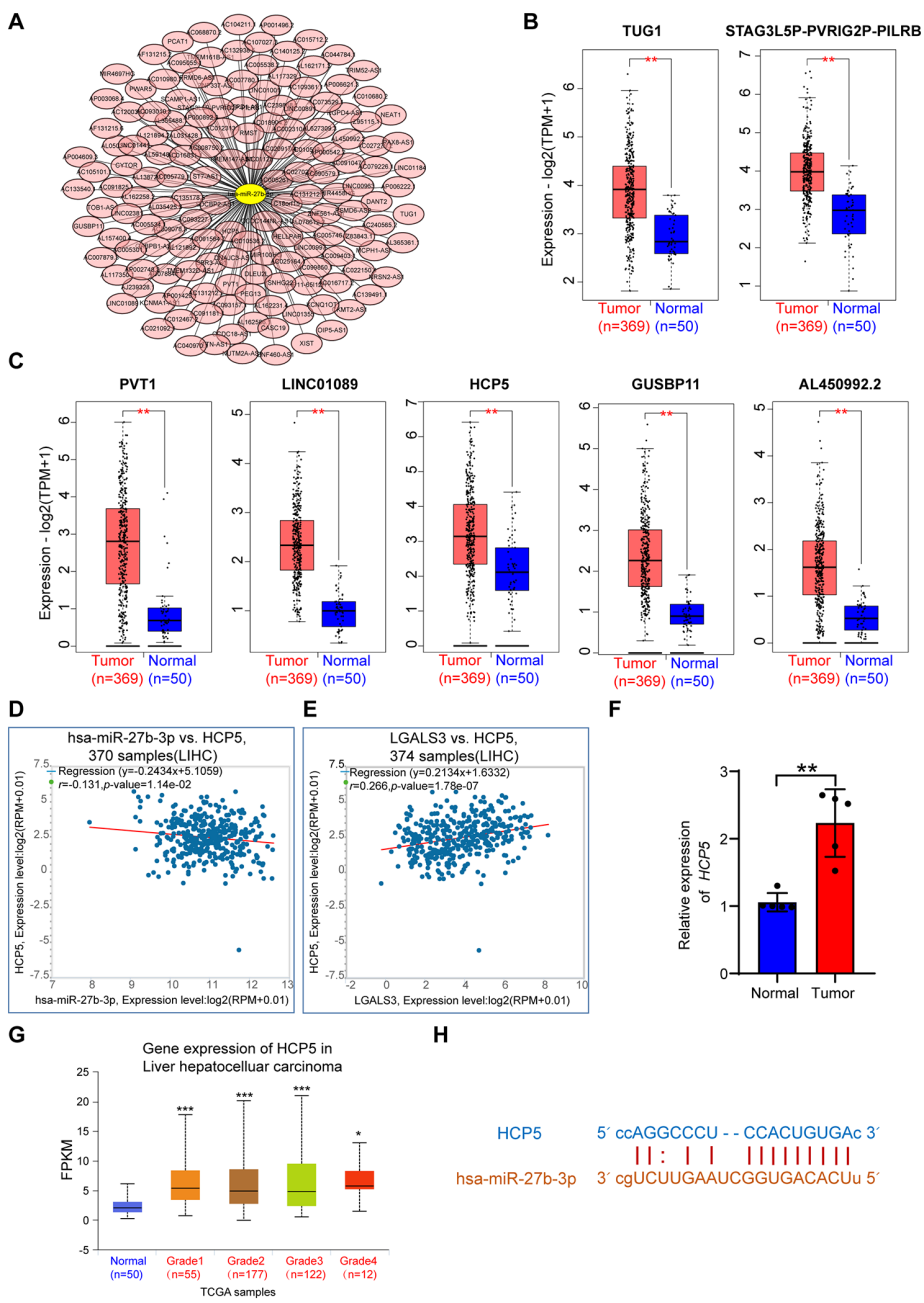
Prediction and identification of the upstream lncRNAs interacting with hsa-miR-27b-3p in HCC. (**A**) Candidate lncRNAs interacting with hsa-miR-27b-3p established by Cytoscape software. (**B**–**C**) The expression of TUG1, STAG3L5P-PVRIG2P-PILRB, PVT1, LINC01089, HCP5, GUSBP11, and AL450992.2 in TCGA HCC compared to “TCGA normal” data. Correlation analysis between HCP5 and hsa-miR-27b-3p (**D**) or HCP5 and LGALS3 (**E**) in HCC analyzed by ENCORI database. (**F**) qRT-PCR analysis of HCP5 expression in tumor and adjacent normal tissues from 5 HCC patients. (**G**) The differential expression of HCP5 in HCC with various tumor grades assessed by UALCAN. (**H**) Schematic representation of the 3′UTR of HCP5 with the predicted target site for hsa-miR-27b-3p. *p value＜ 0.05 ; **p value < 0.01; ***p value < 0.001

Utilizing ENCORI datasets, the upstream lncRNAs of hsa-miR-27b-3p were forecasted. Findings showed that 153 participant lncRNAs bound to hsa*-*miR*-*101*-*3p were forecasted. Cytoscape software was established for improving visualization by constructing a lncRNA-hsa-miR-27b-3p regulatory network (Fig. 4A). Then, we evaluated predicted lncRNAs expression levels among HCC and corresponding normal tissues utilizing GEPIA2. Among all the 153 lncRNAs, only TUG1, STAG3L5P-PVRIG2P-PILRB, PVT1, LINC01089, HCP5, GUSBP11, and AL450992.2 were markedly up-regulated in HCC (Fig. 4B–C). LncRNAs can serve as competing for endogenous RNAs (ceRNAs) by binding to shared miRNAs in a competitive manner, thereby enhancing mRNA expression [16]. Hence, the association between candidate lncRNAs and LGALS3 should be positive, whereas the association between lncRNAs and hsa-miR-27b-3p should be negative. Figure 4D suggests that only HCP5 had a significant inverse relationship with hsa-miR-27a-3p in HCC. No statistical expression relationships among hsa-miR-27b-3p and the other six predicted lncRNAs were observed (data not shown). Meanwhile, we detected a significant positive association between HCP5 and LGALS3 in HCC (Fig. 4E). Subsequently, HCP5 expression was validated by qRT–PCR assay of tumor and adjacent normal tissues from 5 HCC patients. Here, HCP5 expression was also markedly increased in HCC (Fig. 4F). Moreover, results further demonstrate that HCP5 expression is higher in various grades of HCC in comparison to normal control (Fig. 4G). Figure 4H shows the forecasted interaction between the HCP5 and hsa-miR-27b-3p. According to expression and correlation analyses, HCP5 could be the greatest promising upstream lncRNA of hsa-miR-27b-3p/LGALS3 axis within HCC.

### Immune cell infiltration and cell chemotaxis analysis of LGALS3 within HCC

**Fig. 5:**
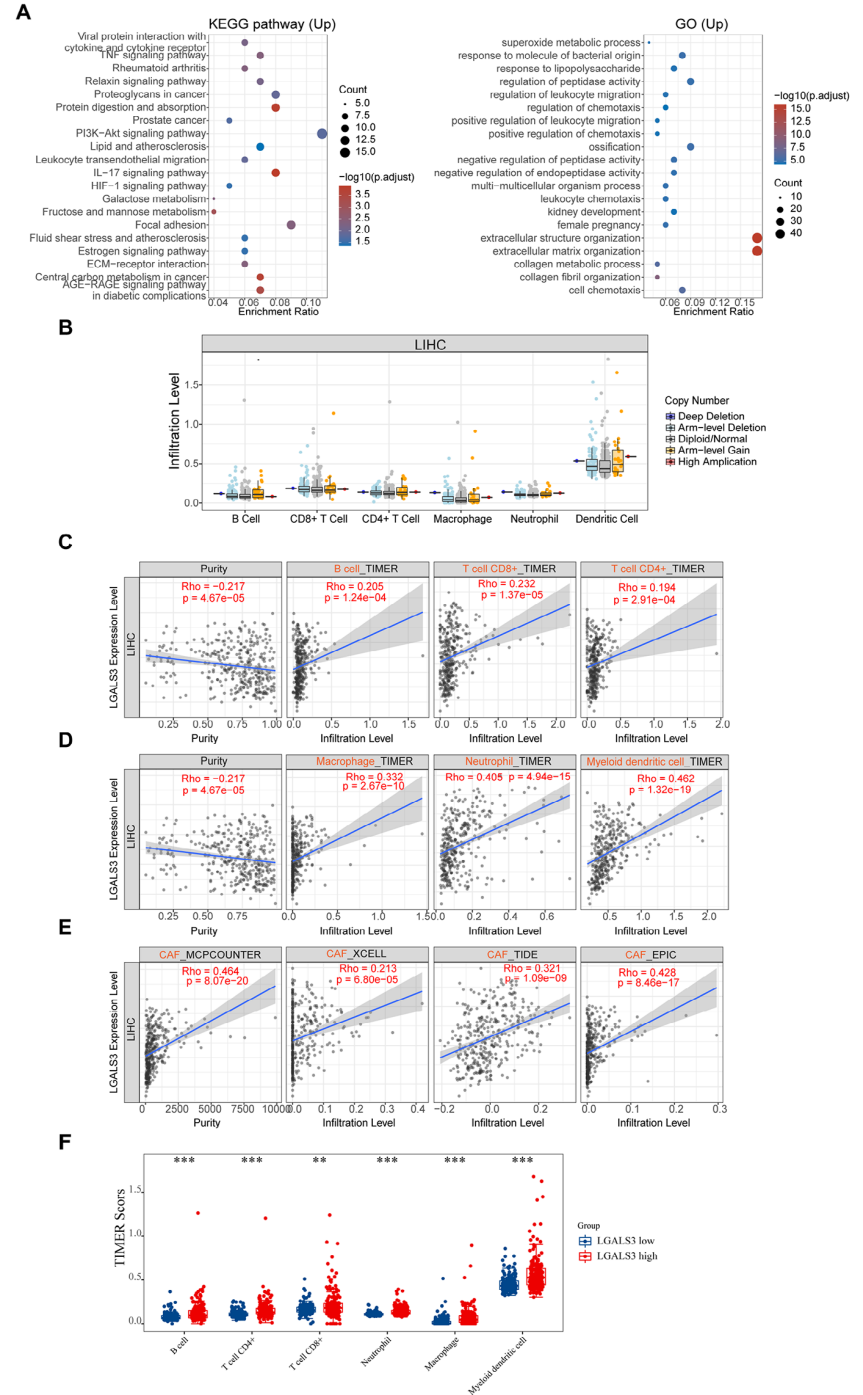
The relationship of LGALS3 expression with immune cell infiltration in HCC. (**A**) The enriched KEGG signaling pathways were selected to demonstrate the biological actions of major potential mRNA. The abscissa indicates gene ratio and the enriched pathways were presented the ordinate. GO analysis of potential targets of mRNAs. Colors represent the significance of differential enrichment, the size of the circles represents the number of genes, the larger the circle, the greater the number of genes. In the enrichment result, *p* < 0.05 is considered to be a meaningful pathway. (**B**) Association of copy number alteration of LGALS3 with the infiltration level of various immune cells in HCC assessed by TIMER. (**C**–**E**) Correlation analysis between LGALS3 expression level and B cell, CD4^+^ T cell, CD8^+^ T cell, neutrophil, macrophage, dendritic cell, or CAFs infiltration level in HCC assessed by TIMER2. (**F**) The expression distribution of immune score in HCC patients with high and low LGALS3 expression. The abscissa represents immune cell types, and the ordinate represents the expression distribution of immune score in low LGALS3 expression groups or high LGALS3 expression groups. **p value < 0.01; ***p value < 0.001

To delineate the driving mechanism of LGALS3 for the malignant progression of HCC, the KEGG and GO analysis was conducted. The volcano plot and heatmap showed significantly differentially expressed genes between LGALS3 high- and low-expression groups (Figure S3A–B). The KEGG pathway analysis revealed that these LGALS3-related genes were enriched in the IL-17 signaling pathway, ECM-receptor interaction pathway, central carbon metabolism in cancer pathway, leukocyte transendothelial migration pathway and PI3K-Akt signaling pathway. Meanwhile, the GO analysis revealed that these genes were strongly associated with cell chemotaxis, leukocyte chemotaxis, regulation of leukocyte migration, as well as regulation of chemotaxis (Fig. 5A). Accumulating evidence has proven the immune system has an essential role in malignancy pathogenesis [17], and LGALS3 is closely correlated with CD163^+^ tumor-associated macrophages (TAM) in glioma [10]. Therefore, we studied the association between LGALS3 level and the immune infiltration level in HCC. There was no statistical difference in immune cell infiltration levels over a number of LGALS3 copy numbers (Fig. 5B). Meanwhile, immune infiltration analysis revealed that expression of LGALS3 showed a significant positive association with several immune cell populations, involving CD4^+^ T cell, CD8^+^T cell, B cell, neutrophil, macrophage, dendritic cell, as well as cancer-associated fibroblasts (CAFs) within HCC (Fig. 5C–E). Based on these results, we further evaluated the immune score in HCC patients with high and low LGALS3 expression. The scores of immune cells, including CD4^+^ T cell, CD8^+^T cell, B cell, neutrophil, macrophage, and dendritic cell, were significantly higher in the high LGALS3 expression groups, as shown in Fig. 5F. Chemokines are a group of molecules that are important for the chemotaxis of immune cells [18]. According to Table S1, the expression of LGALS3 was statistically positively correlated with several chemokines of immune cells, involving monocytes/macrophages (CCL2, CCL3, CCL5, CCL7, CCL13, CCL17, and CCL22), T lymphocytes (CCL2, CCL1, CCL17, and CCL22), eosinophils (CCL11, CCL26, CCL5, CCL7, CCL13, and CCL3), mast cells (CCR1, CCR2, CCR3, CCR4, CCR5, CXCR2, and CXCR4), and neutrophils (CXCL8). Taken together, these outcomes indicate that LGALS3 is positively associated with immune cell infiltration and cell chemotaxis and could have a crucial function in HCC tumor immune microenvironment.

### LGALS3 expression correlation and immune cell biomarkers in HCC

Next, we wanted to investigate the LGALS3 function in HCC tumor immunity further. Utilizing GEPIA databases, we studied the correlation between LGALS3 expression and immune cell biomarkers within HCC. Table S2 lists that LGALS3 demonstrated a significant positive association with various immune cell biomarkers, including B cell (CD19 and CD79A), CD4^+^ T cell (CD4), CD8^+^ T cell (CD8A and CD8B), neutrophil (ITGAM and CCR7), M1 macrophage (NOS2, IRF5, and PTGS2), M2 macrophage (CD163, VSIG4, and MS4A4A), dendritic cell (HLA-DPB1, HLA-DQB1, HLA-DRA, HLA-DPA1, CD1C, NRP1, and ITGAX) and CAFs (FAP, ACTA2, S100A4, PDPN, PDGFR, and CD70) in HCC. Our results indicate that LGALS3 has a significant positive connection to the immune infiltration within HCC, especially CAFs.

### Association between LGALS3 and immunological checkpoints in HCC

**Fig. 6:**
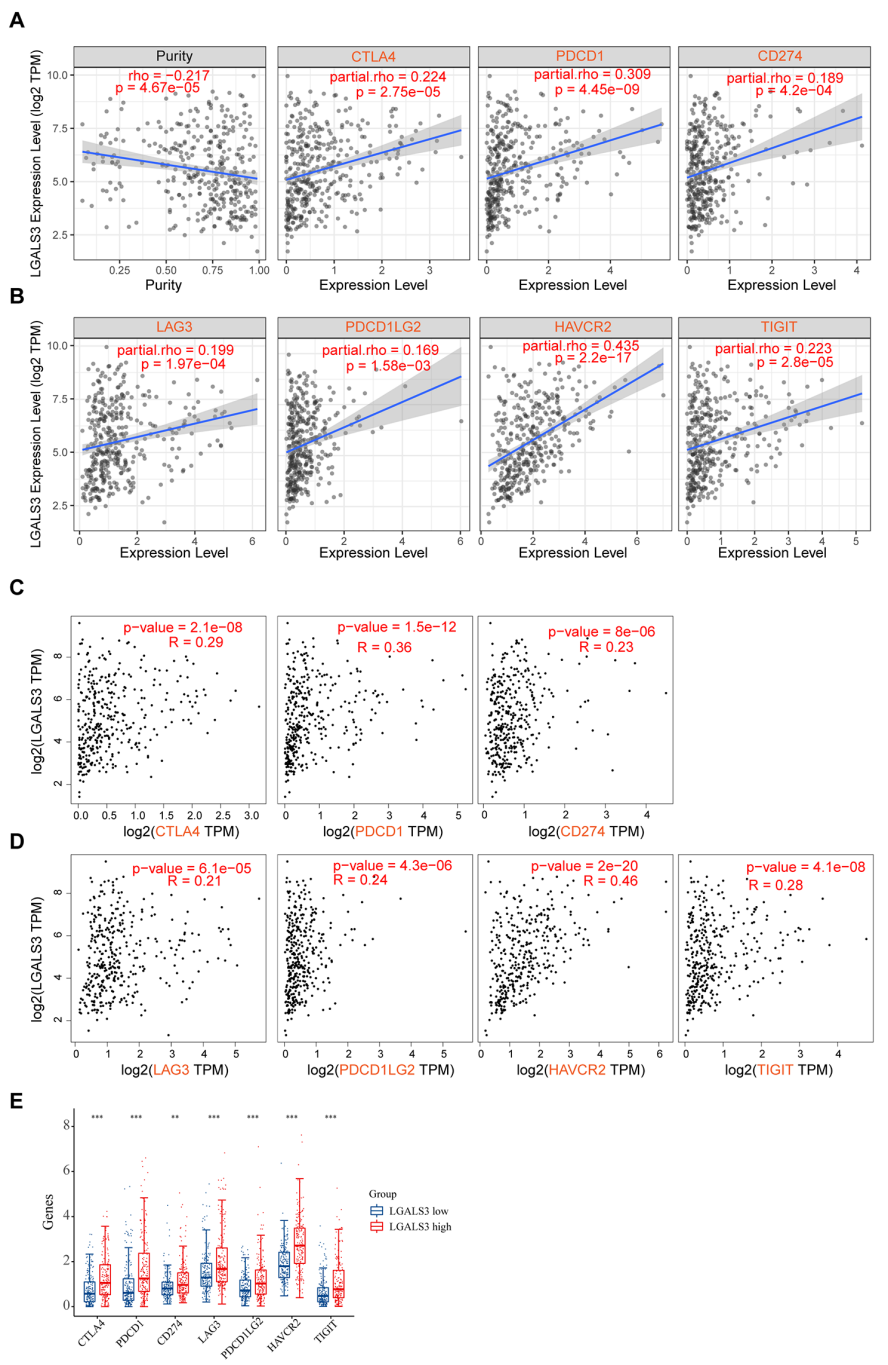
The relationship between LGALS3 expression and immune checkpoint genes in HCC. (**A**–**B**) The correlation of LGALS3 with expression of CTLA4, PDCD1, CD274, LAG3, PDCD1LG2, HAVCR2 or TIGIT in HCC analyzed by TIMER2. (**C**–**D**) The expression correlation of LGALS3 with the expression of CTLA4, PDCD1, CD274, LAG3, PDCD1LG2, HAVCR2, or TIGIT in HCC assessed by GEPIA database. (**E**) The expression distribution of CTLA4, PDCD1, CD274, LAG3, PDCD1LG2, HAVCR2, or TIGIT gene in HCC patients with high and low LGALS3 expression. The abscissa represents different groups of samples, and the ordinate represents the expression distribution of gene, different colors represent different groups. **p value < 0.01; ***p value < 0.001

The immune checkpoint molecules have a vital function in immune surveillance, immune escape, as well as immune editing [19]. To further determine the possible oncogenic function of LGALS3 in HCC, the association between LGALS3 and several immunological checkpoints was evaluated. LGALS3 expression showed a significant positive association with CD274, TIGIT, PDCD1, HAVCR2, CTLA4, LAG3, as well as PDCD1LG2 after adjustment for tumor purity in HCC (Fig. 6A–B). Moreover, based on GEPIA2, the same positive association was identified between LGALS3 and these immune checkpoints (Fig. 6C–D). Furthermore, we found that the expression of these immune checkpoints was significantly upregulated in the high LGALS3 expression groups, as shown in Fig. 6E. These findings imply that tumor immune escape may have a role in LGALS3-mediated HCC carcinogenesis.

### HCP5 is significantly correlated with immune cell infiltrations, cell chemotaxis, and immune checkpoints in HCC

**Fig. 7:**
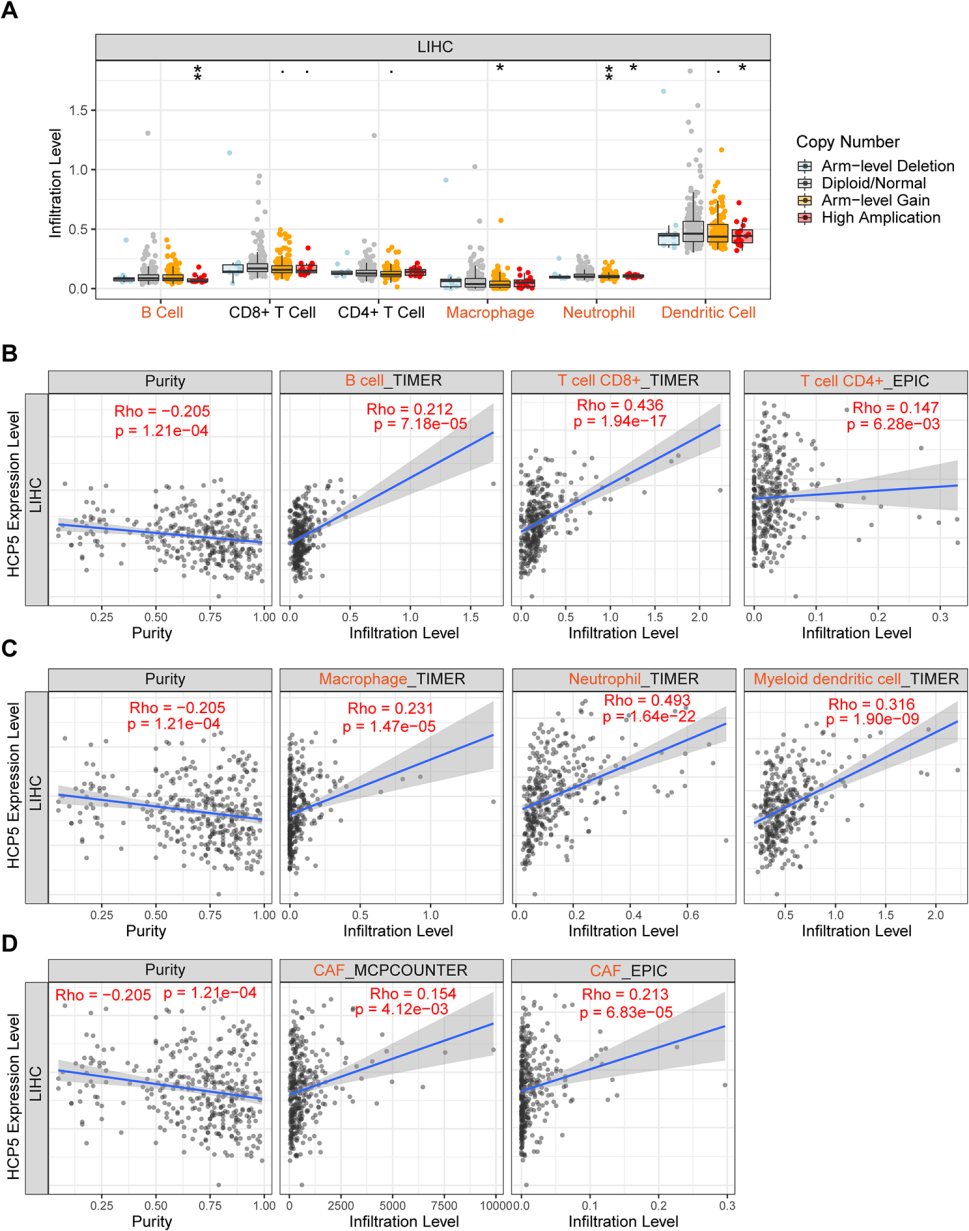
The relationship of HCP5 expression with immune cell infiltration in HCC. (**A**) Association of copy number alteration of HCP5 with the infiltration level of various immune cells in HCC assessed by TIMER. *p value ＜0.05; **p value ＜ 0.01.  (**B**–**D**) Correlation analysis between HCP5 expression level and B cell, CD4^+^ T cell, CD8^+^ T cell, neutrophil, macrophage, dendritic cell, or CAF infiltration level in HCC assessed by TIMER2

HCP5 expression was abnormally high in many cancer types and its dysregulation appears closely linked to progression [20]. Next, we wanted to investigate the HCP5 role in tumor immunity of HCC. Figure 7A shows that HCC immune cell infiltration levels, including macrophages, neutrophils, B cells, and dendritic cells, may be influenced by HCP5 copy number variations. Meanwhile, we detected a significant positive association between expression of HCP5 and B cell, CD4^+^ T cell, CD8^+^T cell, neutrophil, CAFs, dendritic cell, as well as macrophage infiltration levels within HCC **(**Fig. 7B–D**)**, which was further confirmed by corresponding immune biomarkers (Table S3). Table S4 presents that HCP5 was statistically positively associated with various related chemokines of immune cells, including monocytes/macrophages, T lymphocytes, eosinophils, mast cells, and neutrophils. Moreover, expression of HCP5 was statistically positively associated with the above-mentioned immunological checkpoints based on both TIMER2 and GEPIA2 (Figure S4A–D). Taken together, our results indicate that HCP5 is positively associated with immune cell infiltration, cell chemotaxis, as well as immunological checkpoints and may exert an immunoregulatory role in HCC.

### Correlations between HCP5/hsa-miR-27b-3p/LGALS3 axis and pathways in HCC

**Fig. 8:**
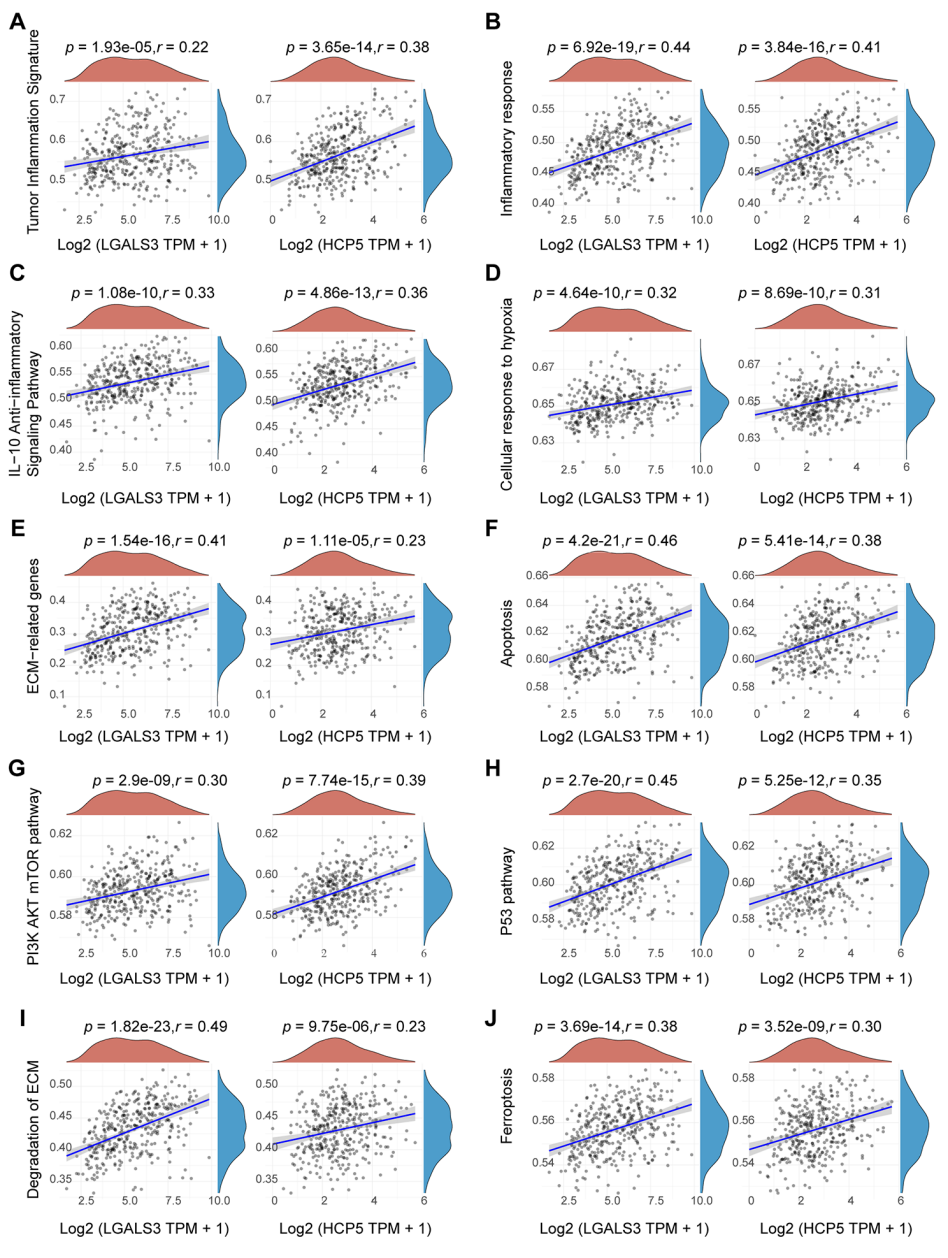
The correlation between LGALS3 or HCP5 and pathway score in HCC analyzed by Spearman method. The abscissa represents the expression of LGALS3 or HCP5 gene, and the ordinate represents the pathway score of this gene in HCC. The red curve represents the density distribution of LGALS3 or HCP5 gene, and the blue curve represents the distribution of pathway score. The positive correlation between LGALS3 or HCP5 and pathway score are given (|*r*| >0.2, *p* value < 0.05, *n* = 371). Ten pathways are included: (**A**) tumor inflammation signature; (**B**) inflammatory response; (**C**) IL-10 anti-inflammatory signaling pathway; (**D**) cellular response to hypoxia; (**E**) ECM-related genes; (**F**) apoptosis; (**G**) PI3K/AKT/mTOR pathway; (**H**) P53 pathway; (**I**) degradation of ECM; (**J**) ferroptosis

To explore how HCP5/hsa-miR-27b-3p/LGALS3 axis took part in the incidence of malignant progression of HCC, we performed the correlation analysis between HCP5 or LGALS3 gene and pathway scores based on the gene set variation analysis (GSVA) analysis. As expected, inflammatory response, tumor inflammation signature score, and IL-10 anti-inflammatory signaling pathway were positively correlated with HCP5 and LGALS3 in HCC (Fig. 8A–C), implying that the high level of HCP5 or LGALS3 expression is characterized by a high immune cell infiltration. Meanwhile, the GSVA analysis indicated that expression of HCP5 and LGALS3 is positively associated with multiple pathways, involving cellular response to hypoxia, ECM-related genes, apoptosis, PI3K/AKT/mTOR pathway, P53 pathway, degradation of ECM, and ferroptosis (Fig. 8D–J). It is well established that tumor cell metabolism has a crucial function in tumor immune escape [21]. Meanwhile, the expression of HCP5 and LGALS3 was found to be positively associated with nine metabolism-related pathways, including the amino sugar and nucleotide sugar metabolism, inositol phosphate metabolism, glycerophospholipid metabolism, ether lipid metabolism, sphingolipid metabolism, glycosphingolipid biosynthesis ganglio series, glycosaminoglycan biosynthesis keratan sulfate, phosphonate and phosphinate metabolism, as well as neomycin kanamycin and gentamicin biosynthesis, and negatively associated with four metabolism-related pathways, including steroid biosynthesis, arginine biosynthesis, D Arginine and D ornithine metabolism, and biotin metabolism. (Figure S5A–M). These findings suggest the possible signaling pathway linked to the HCP5/hsa-miR-27b-3p/LGALS3 axis in immune evasion and metabolic reprogramming of HCC.

## Discussion

LGALS3 has a crucial function in mediating cell adhesion as well as cell-cell interaction by recognizing complex carbohydrates on the surface of cells [22, 23], as well as regulates cell apoptosis, autophagy, and inflammation [24, 25]. Interestingly, recent studies suggested that LGALS3 involves in essential cancer-related mechanisms, including cellular metabolism, carcinogenesis, metastasis, neoplasia, angiogenesis, as well as immune escape [26–28]. In addition, LGALS3 is highly expressed and implicated in different cancer types progression such as HCC, gastric, colorectal, pancreatic carcinomas, melanomas or glioblastomas and breast cancer [29, 30]. Indeed, LGALS3 has been considered a potential marker for these malignancies. Interestingly, LGALS3, which is differentially expressed in different cancers, has been shown to exhibit tumor suppressor activity in certain cancer types. The different roles of LGALS3 may be attributed to different potential mechanisms that appear cancer-type dependent. The different locations and mutations of LGALS3 also contribute to its various functions. However, LGALS3 remains inadequately understood in HCC and requires further investigation.

First, we conducted an extensive investigation of the expression profile, clinical prognosis, and pathologic stage of LGALS3 in HCC through an in-depth analysis of the public database. On the basis of TCGA and CPTAC datasets, we found that LGALS3 gene and protein expression was elevated in HCC tissues. Moreover, the OS and DSS were lesser in patients with HCC having higher expression levels of LGALS3 contrasted to those with low expression levels of LGALS3 based on GEPIA2 and Kaplan-Meier plotter datasets. Meanwhile, the expression of LGALS3 within HCC was significantly associated with the advanced tumor stage and grade, indicating that elevated LGALS3 expression could increase tumor progression. Song et al. [31] indicated that galectin-3 promoted HCC tumorigenesis and metastasis via β-catenin signalling in vitro and in vivo. Cao et al. [32] suggested that upregulation of LGALS3 enhanced cell migration, invasion, and EMT of HCC. These studies, along with our findings, suggested that LGALS3 plays a key role in hepatocarcinogenesis and malignant progression.

NcRNAs, consisting of miRNAs, circular RNAs (circRNAs), and lncRNAs, participate in various aspects of cancer biology, involving cell death, proliferation, and treatment resistance [33]. In order to determine the miRNAs that regulate LGALS3, we first predicted the possible potential miRNAs of LGALS3 using the ENCORI database and finally found 12 miRNAs. Subsequently, hsa-miR-27b-3p was determined as a prospective upstream tumor suppressive miRNA of LGALS3 according to correlation and expression analysis. We then predicted upstream lncRNAs of the hsa-miR-27b-3p/LGALS3 axis, and identified 153 potential lncRNAs. According to ceRNA hypothesis, correlation and expression analysis, HCP5 was selected as the upstream lncRNA of hsa-miR-27b-3p. It has been well documented that HCP5 functions as oncogenes in various types of cancer, involving HCC [34], GEM [35] and clear cell RCC [36]. For instance, HCP5 facilitated HCC cell proliferation, metastatic and invasive capacity through miR-29b-3p/DNMT3A/AKT axis [34]. Taken together, we predict that HCP5/hsa-miR-27b-3p axis could be an important regulatory mechanism of LGALS3 in HCC.

Recent studies gradually began to recognize the importance of the TME, which has a determinative function in tumor survival, progression, as well as metabolic activities [37, 38]. CAFs and tumor immune cell infiltrations of the TME have been linked to a prognosis for cancer patients and the efficacies of various therapies, including chemotherapy, radiotherapy, as well as immunotherapy [39, 40]. Immune infiltration patterns of HCC can be evaluated to gain a deeper understanding of the TME and to establish new avenues for individualized immunotherapy strategies [41]. The immune cell infiltration in the TME was inextricably linked with the progression of HCC. For example, CD4^+^T cells, CD8^+^T cells, M1 macrophages, B cells, and memory T cells might be associated with favorable prognosis in HCC, while regulatory T cells, regulatory B cells, and M2 macrophages might be related to the poor prognosis in HCC. Moreover, the excessive infiltration of stromal components within tumor tissues impedes the migration of immune cells to the tumor site, indicating that the intercellular communication within TME may have a greater impact on tumor initiation and progression than individual cell populations. Our findings indicated that both LGALS3 and HCP5 were significantly positively correlated with several immune cell populations as well as their biomarkers, including CD4^+^ T cell, CD8^+^T cell, neutrophil, macrophage, B cell, dendritic cell, as well as CAFs in HCC. Moreover, LGALS3 and HCP5 were significantly positively linked to multiple related chemokines of immune cells, including monocytes/macrophages, T lymphocytes, eosinophils, mast cells, and neutrophils. Previous studies revealed that eight genes related to M2-like TAM, including LGALS3, develop a novel prognostic signature and uncover the HCC immune landscape [42]. Liu et al. indicated that HCP5 might act as a ceRNA to modulate MAD2L1, which is associated with tumor immune infiltration as well as poor prognosis in HCC [43]. These studies, together with our results, showed that tumor immune infiltration could contribute to HCP5/hsa-miR-27b-3p/LGALS3 axis-mediated malignant progression and poor prognosis of HCC.

In addition to the tumor immune cell infiltration of the TME, immunotherapy efficacy is associated with the expression of immune checkpoints [44, 45]. Immune checkpoint inhibitors show varying effectiveness and side effects in different patients, highlighting the need to identify those who may benefit from this therapy. In the current research, the LGALS3 and HCP5 expression levels were significantly positively associated with multiple immune checkpoints, which provided potential targets for immunotherapy and indicated a better response to the immune-inhibiting treatments in HCC patients with high LGALS3 and HCP5 expression. Our results also showed that these immune checkpoints were significantly upregulated in the high LGALS3 expression groups. However, CD4^+^ T cells, CD8^+^T cells, and B cells which play an antitumor role were higher in the high LGALS3 expression groups. These results indicate that LGALS3 expression may have multiple effects on the immune microenvironment. We believe that LGALS3 could be considered as a potential target for therapy. However, direct targeting of LGALS3 may have various off target effects due to its positive correlation with immune infiltration. Therefore, a good understanding of the LGALS3’s regulatory mechanisms, its downstream effectors, and its role in tumor immune microenvironment is of great importance for alone or in combination treatment in HCC. Previous studies revealed that in solid tumor patients, blocking LGALS3, a ligand for LAG3, in conjunction with immune approaches may promote anti-tumor immunity and boost tumor regression [9]. Numerous studies have confirmed that ncRNA can regulate multiple immune checkpoints to influence tumor proliferation, differentiation, and development [46]. Taken together, our findings indicated that targeting LGALS3 and HCP5 might improve the efficacy of immunotherapy in HCC.

Abnormal energy metabolism has been recognized as one of the characteristics of tumors [47]. HCC is characterized by a deregulation of cellular energetics involving an increase in glycolysis [26]. LGALS3 has been established to have a crucial function in glycolysis and contributes to tumor metabolic reprogramming to adapt to oxygen and nutrient deprivation in TME [26]. Previous studies revealed that LGALS3 as a key regulator of glucose metabolism promoted the progression of HCC by stimulating the mTORC1 signaling [48]. In addition to these studies, our findings provide new information. Specifically, we revealed several potential signaling pathways linked to the HCP5/hsa-miR-27b-3p/LGALS3 axis in immune evasion and metabolic reprogramming of HCC. However, signaling pathways involving the HCP5/hsa-miR-27b-3p/LGALS3 axis still need to be confirmed through further experimental studies.

## Conclusions

**Fig. 9:**
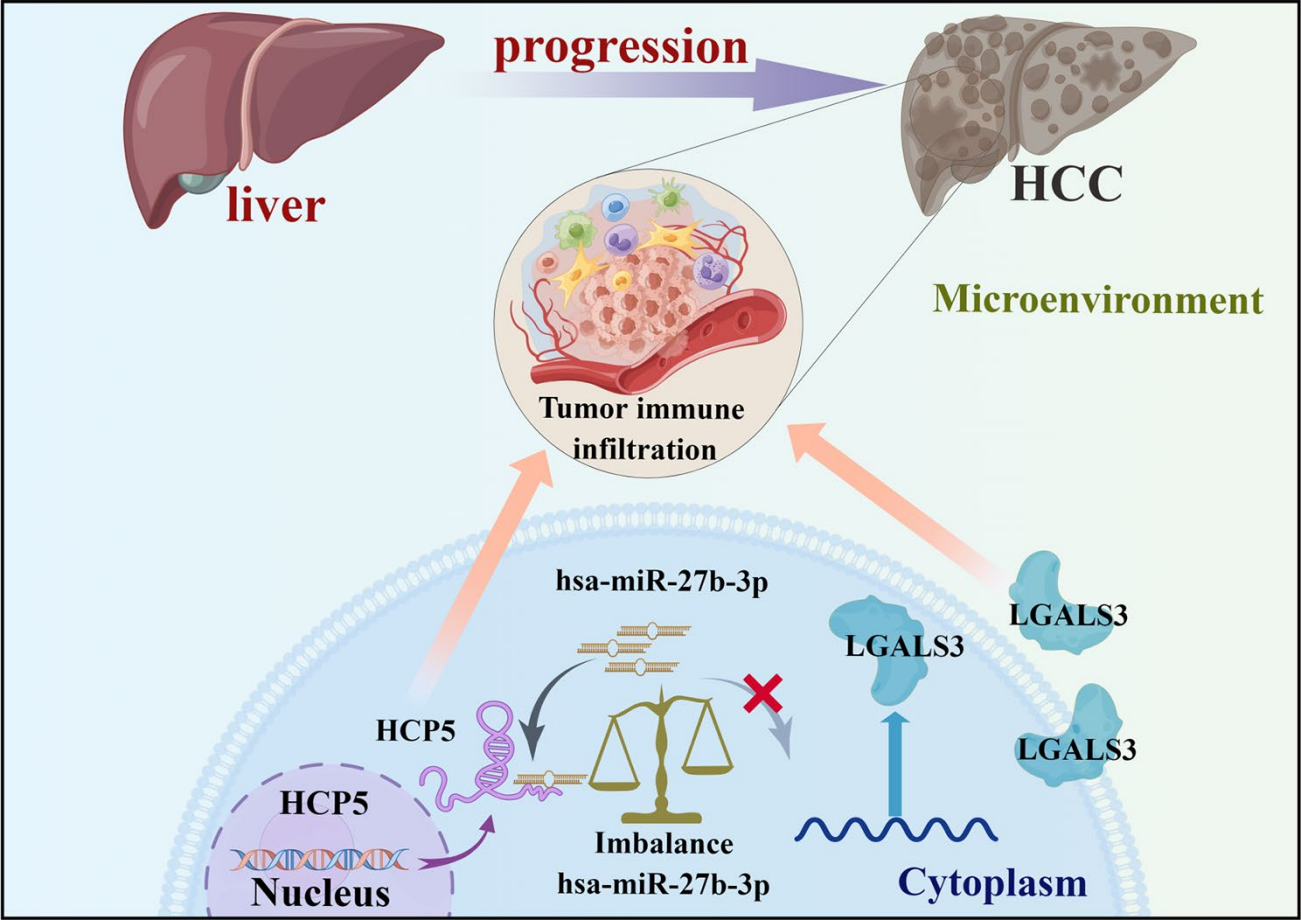
The model of HCP5/hsa-miR-27b-3p/LGALS3 axis in carcinogenesis of HCC (By Figdraw)

In conclusion, LGALS3 is greatly expressed in HCC, and its expression is associated with poor patient prognosis and immune cell infiltration. Moreover, our current discoveries suggest that LGALS3 may play a pivotal role in promoting hepatocarcinogenesis and malignant progression by enhancing tumor immune cell infiltration and upregulating immune checkpoint expression. Investigating the correlation between LGALS3 and immune cells has the potential to improve immunotherapy and tumor treatment. A novel HCP5/hsa-miR-27b-3p axis may be a key epigenetic mechanism that regulates LGALS3 expression and immune cell infiltration, suggesting that LGALS3 could be a promising treatment target and prognostic biomarker for HCC (Fig. 9). Additionally, our findings provide insight into molecular mechanisms related to the HCP5/hsa-miR-27b-3p/LGALS3 axis in HCC. One limitation of our study was that no additional experiments were conducted to verify the bioinformatics analysis’s findings. Further basic research and large clinical trials will be planned to resolve this issue.

## Materials and methods

### Gene expression analysis

TIMER2 [49] (Tumor Immune Estimation Resource, version 2, http://timer.cistrome.org/) was utilized to compare the LGALS3 differential expression among tumors and nearby normal tissues throughout various tumor types of the TCGA project. For specific kinds of cancer in TCGA project without matching normal tissues, we utilized the GEPIA2 [50] (Gene Expression Profiling Interactive Analysis, version 2, http://gepia2.cancer-pku.cn/#analysis) to evaluate the differential expression of LGALS3 among these tumors and normal tissues of the GTEx database. The GEPIA was also employed to estimate the predicted lncRNA expression in HCC. The selection criteria were set as *p*-value < 0.01 for identifying statistically significant. The bioinformatics portal UALCAN [51] (http://ualcan.path.uab.edu/analysis-prot.html) offers an option for protein expression analysis employing CPTAC data. Hence, we investigated the LGALS3 total protein expression level in primary tumor and normal tissues. The available datasets of nine tumors among the 23 types of cancer were chosen, namely, clear cell RCC, GBM, breast cancer, LIHC, colon cancer, HNSC, LUAD, LUSC as well as UCEC. Besides, the UALCAN portal was employed to analyze the hsa-miR-27b-3p and HCP5 expression in different pathological stages or grades of HCC.

### Tissue samples and immunofluorescence

We collected pairs of resected tumor tissues and adjacent nontumor tissues from 5 HCC patients who underwent surgery at Tianjin First Central Hospital. Detailed characteristics of our patients are provided in Supplementary Information, Table S6. Following standard procedures, the tissue samples were subjected to fixation in a 4% formalin solution for 24 h, followed by embedding in paraffin. Tissue sections were incubated at 4 °C overnight with primary antibody against LGALS3 (GB12940, Servicebio, 1:500 dilution). Subsequently, samples were incubated with Cy3-conjugated goat anti-mouse IgG secondary antibody (GB21301, Servicebio, 1:300 dilution). Immunofluorescence images were performed using a Nikon fluorescence microscope (Tokyo, Japan).

### Quantitative PCR (qPCR)

Trizol reagent (Invitrogen) was employed to extract total RNA from frozen tissue samples. The RNA was subjected to reverse transcription to generate cDNA using the PrimeScript RT reagent kit (Invitrogen). qPCR was carried out using the Power SYBR Green Master Mix (ABI, USA) following the manufacturer’s recommendations. GAPDH or U6 acted as endogenous control. Primers of genes were as follows: LGALS3 forward: 5ʹ-GCCACTGATTGTGCCTTATAACC-3ʹ and reverse: 5ʹ-AAAACCGACTGTCTTTCTTCCC-3ʹ; HCP5 forward: 5ʹ- AGATTCTCCCCAGACGCCAA-3ʹ and reverse: 5ʹ- CTTGCATCTCAGTCTATTGCCTTT-3ʹ; GAPDH forward: 5ʹ- GGAAGCTTGTCATCAATGGAAATC-3ʹ and reverse: 5ʹ- TGATGACCCTTTTGGCTCCC-3ʹ; hsa-miR-27b-3p RT primers: 5ʹ- CTCAACTGGTGTCGTGGAGTCGGCAATTCAGTTGAGGCAGAACT-3ʹ; hsa-miR-27b-3p forward: 5ʹ-ACACTCCAGCTGGGTTCACAGTGGCTAAG-3ʹ and reverse: 5ʹ-TGGTGTCGTGGAGTCG-3ʹ; U6 forward: 5ʹ-CTCGCTTCGGCAGCACA-3ʹ and reverse: 5ʹ-AACGCTTCACGAATTTGCGT-3ʹ.

### Prognostic analysis of LGALS3

The OS significance map of LGALS3 in the 23 types of cancer was obtained using GEPIA2. The LGALS3 gene median expression level was utilized as a cut-off value. Additionally, we employed the Kaplan-Meier “Survival Analysis” module of GEPIA2 to produce survival plots for the hypothesis test using the log-rank test. Kaplan-Meier plotter (http://kmplot.com/analysis/) database was utilized to examine the influence of LGALS3 on DSS in HCC patients. The “Stage Plot” module of GEPIA2 was processed to create the violin plots of LGALS3 at different pathological stages of HCC. From the TCGA dataset (https://portal.gdc.com), 371 HCC patients’ RNA-sequencing expression profiles (level 3) and related clinical data were retrieved. The GTEx V8 datasets were downloaded from the GTEx Portal Datasets page (https://www.gtexportal.org/home/datasets). To identify independent prognostic factors affecting OS and DSS of HCC patients, the multivariate and univariate Cox regression analyses were employed. Using the R package rms, we analyze the OS and DSS of HCC patients at 1,3, and 5 years and construct a prognostic nomogram. The R package timeROC (v 0.4) was utilized to assess the predictive accuracy of LGALS3. R statistical software packages (R version 4.0.3) were utilized in all statistical analyses and graphics.

### The analysis of candidate miRNA and lncRNAs of LGALS3

ENCORI [52] (Encyclopedia of RNA Interactomes, https://starbase.sysu.edu.cn/index.php) is a database for investigating miRNA-related research. We used the “miRNA-Target” module of ENCORI to speculate the LGALS3 upstream binding miRNAs and candidate lncRNAs that may serve as a sponge to inhibit hsa-miR-27b-3p. Numerous target gene prediction tools, such as PITA, RNA22, miRmap, microT, miRanda, PicTar, as well as TargetScan, anticipated the upstream miRNAs of LGALS3. In addition, we utilized the “Pan-Cancer” module of ENCORI to analyze miRNA differential expression levels, miRNA-LGALS3 co-expression, as well as linRNA-LGALS3 co-expression.

### Immune cell chemotaxis, immune cell infiltration, and immune checkpoint

We applied the “SCNA” module of TIMER (https://cistrome.shinyapps.io/timer/) to assess the association between various copy number variants of LGALS3 or HCP5 and immune cell types within HCC. In addition, we used TIMER2 to analyze the correlation between LGALS3 or HCP5 with immune checkpoint and immune cell infiltration within HCC. We also checked the correlation between LGALS3 or HCP5 with immune cell biomarkers, immune cell chemotaxis, and immune checkpoint using GEPIA2. The RNA-sequencing expression profiles (level 3) of 371 HCC patients were obtained from the TCGA dataset. In order to assess the reliable results of immune score evaluation in HCC patients with high and low LGALS3 expression, we employed the immuneeconv R software package, which integrates the TIMER algorithm. To evaluate the immune checkpoint expression, we utilized the ggplot2 R software package. R statistical software packages (R version 4.0.3) were utilized in all statistical analyses and graphics.

### Functional analysis of LGALS3 and HCP5

From the TCGA dataset, 371 HCC patients’ RNA-sequencing expression profiles (level 3) were retrieved. Using the limma package in the R software, we study the differentially expressed genes between LGALS3 high- and low-expression groups in HCC. To better explore the carcinogenesis of LGALS3, the GO function and KEGG pathway enrichment analysis was conducted using the R package ClusterProfiler (v 3.18.0). Next, the R software GSVA package was utilized for analysis, according to the ssGSEA algorithm [53]. The correlation between LGALS3 or HCP5 and pathway scores, was studied via Spearman correlation. R statistical software packages (R version 4.0.3) were utilized in all statistical analyses and graphics. The selection criteria were set as *p* value < 0.05 and |*r*| > 0.2 for identifying statistically significant.

### Electronic supplementary material

Below is the link to the electronic supplementary material.

Supplementary Material 1

## Data Availability

No datasets were generated or analysed during the current study.
