# Supplementary material for: High LGALS3 expression induced by HCP5/hsa-miR-27b-3p correlates with poor prognosis and tumor immune infiltration in hepatocellular carcinoma
Source: Cancer Cell Int. 2024 Apr 20;24:142. doi: 10.1186/s12935-024-03309-1 (PMC11031979; doi:10.1186/s12935-024-03309-1)
Supplement: Supplementary file 1 — Supplementary Material 1 [file 12935_2024_3309_MOESM1_ESM.docx]

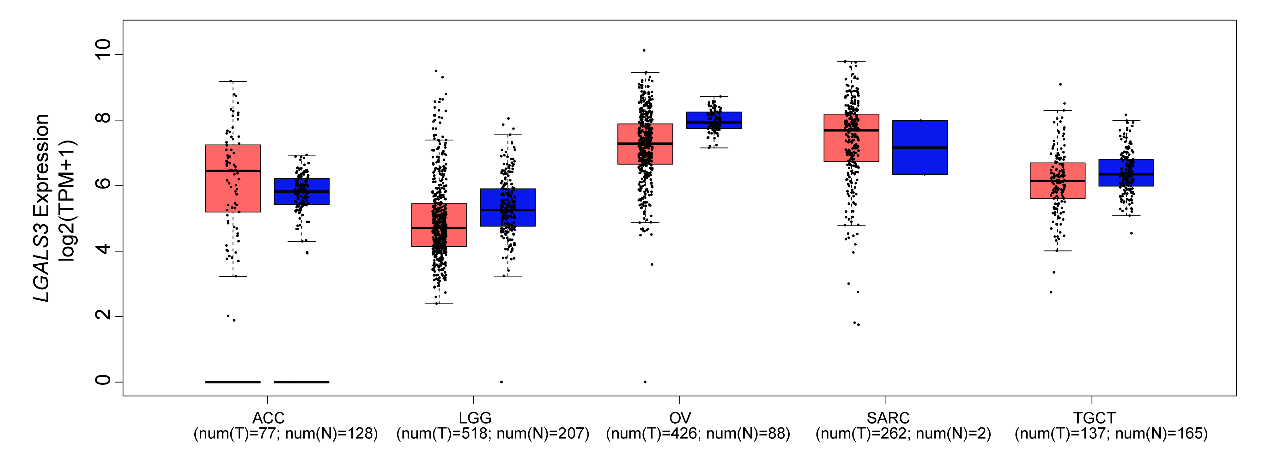


**Figure S1**. Expression level of LGALS3 gene in different tumors

For the type of ACC, LGG, OV, SARC, and TGCT in the TCGA database, the normal control samples of the GTEx database were included as controls.


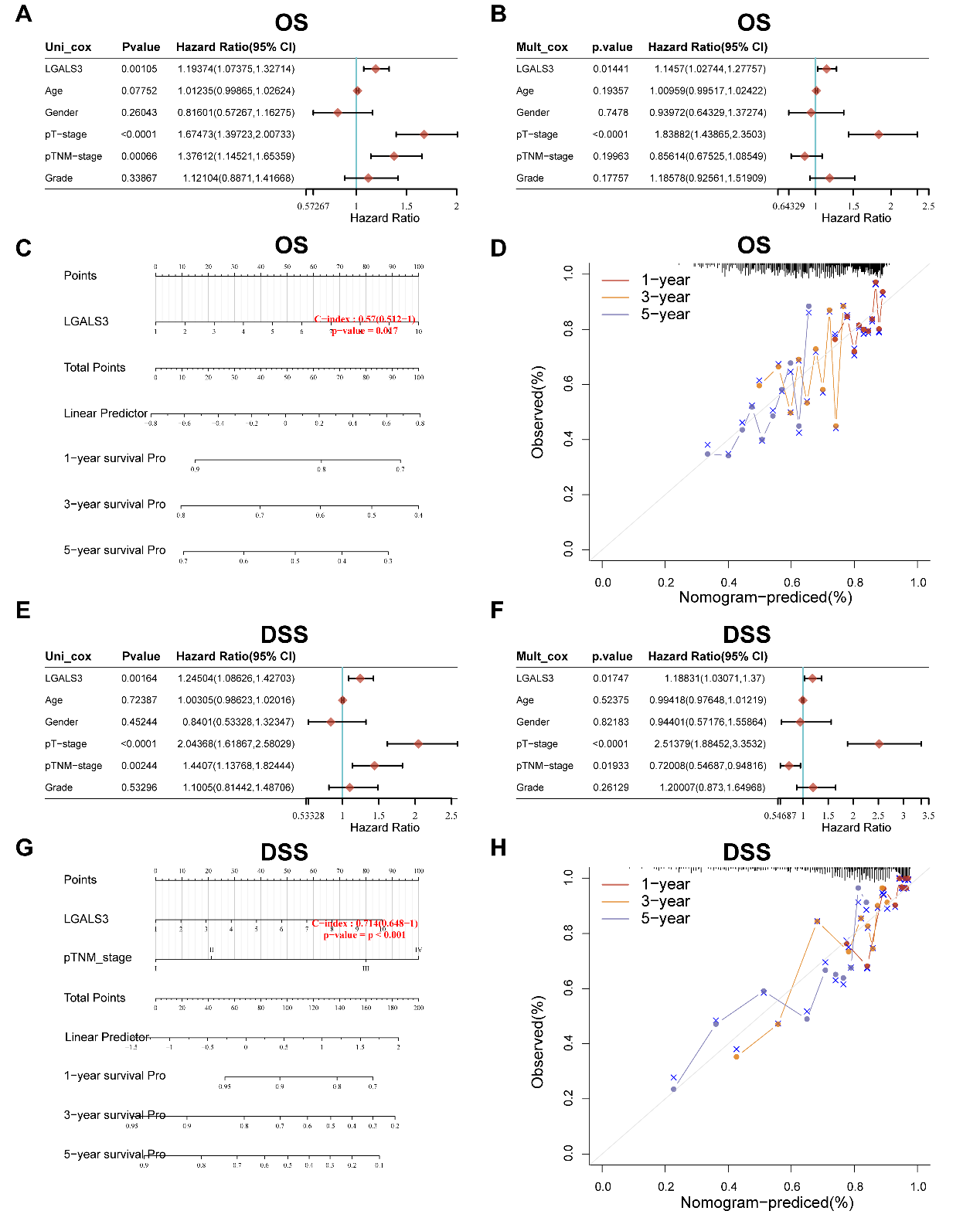


**Figure S2**. LGALS3 was an independent prognostic factor by univariate and multivariate analyses based on OS and DSS

(A) Univariate Cox analysis for OS. (B) Multivariate Cox analysis for OS. (C) The nomogram for OS in patients with HCC. (D) Calibration plot of OS at 1, 3, and 5 years. (E) Univariate Cox analysis for DSS. (F) Multivariate Cox analysis for DSS. (G) The nomogram for DSS in patients with HCC. (H) Calibration plot of DSS at 1, 3, and 5 years.


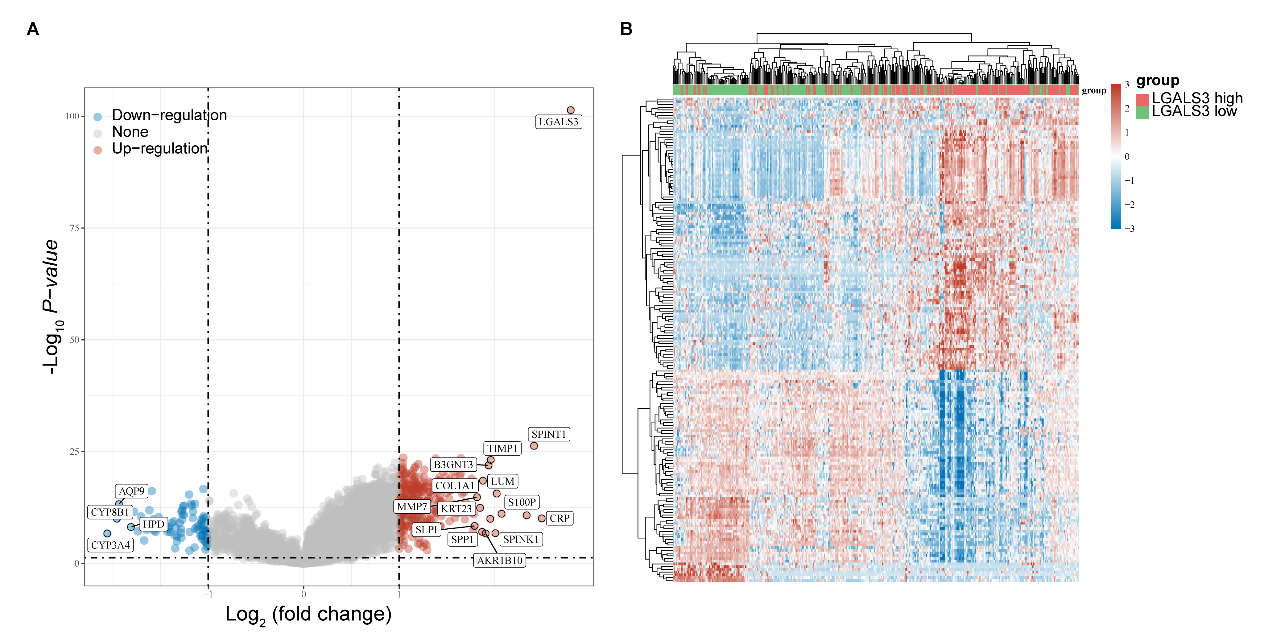


**Figure S3**. Differential gene expression profiles between LGALS3 high- and low-expression groups

(A) Volcano plot highlighting differentially expressed genes. Red dots indicate upregulated genes; blue dots indicate downregulated genes; grey dots indicate not significant. (B) The heatmap of the differential gene expression between LGALS3 high- and low-expression groups. Different colors represent the trend of gene expression in different tissues. The top 50 up-regulated genes and top 50 down-regulated genes were showed in this figure.


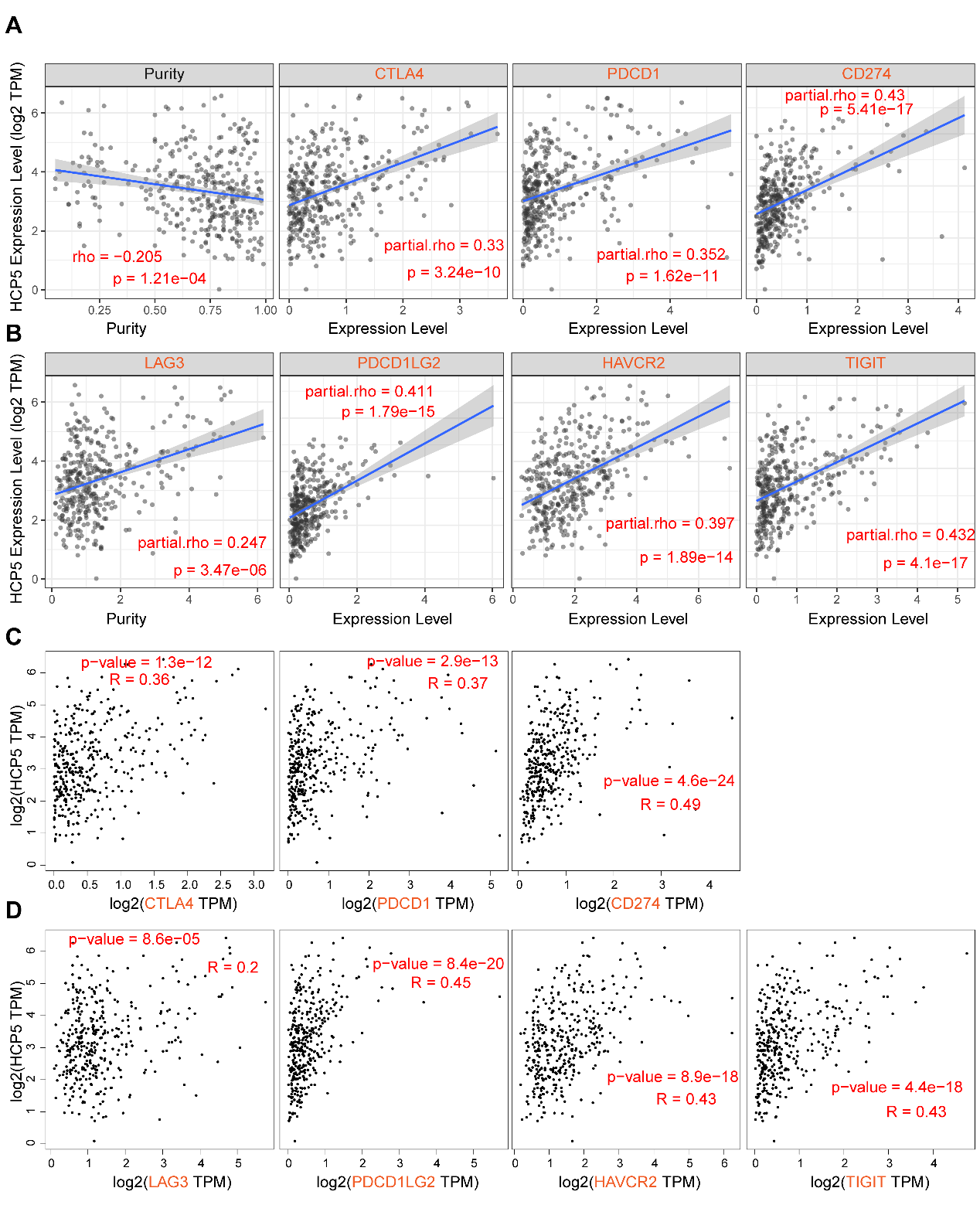


**Figure S4**. The relationship between HCP5 expression and immune checkpoint genes in HCC

(A–B) The correlation of HCP5 with expression of CTLA4, PDCD1, CD274, LAG3, PDCD1LG2, HAVCR2, or TIGIT in HCC analyzed by TIMER2. (C–D) The expression correlation of HCP5 with expression of CTLA4, PDCD1, CD274, LAG3, PDCD1LG2, HAVCR2, or TIGIT in HCC assessed by GEPIA database.


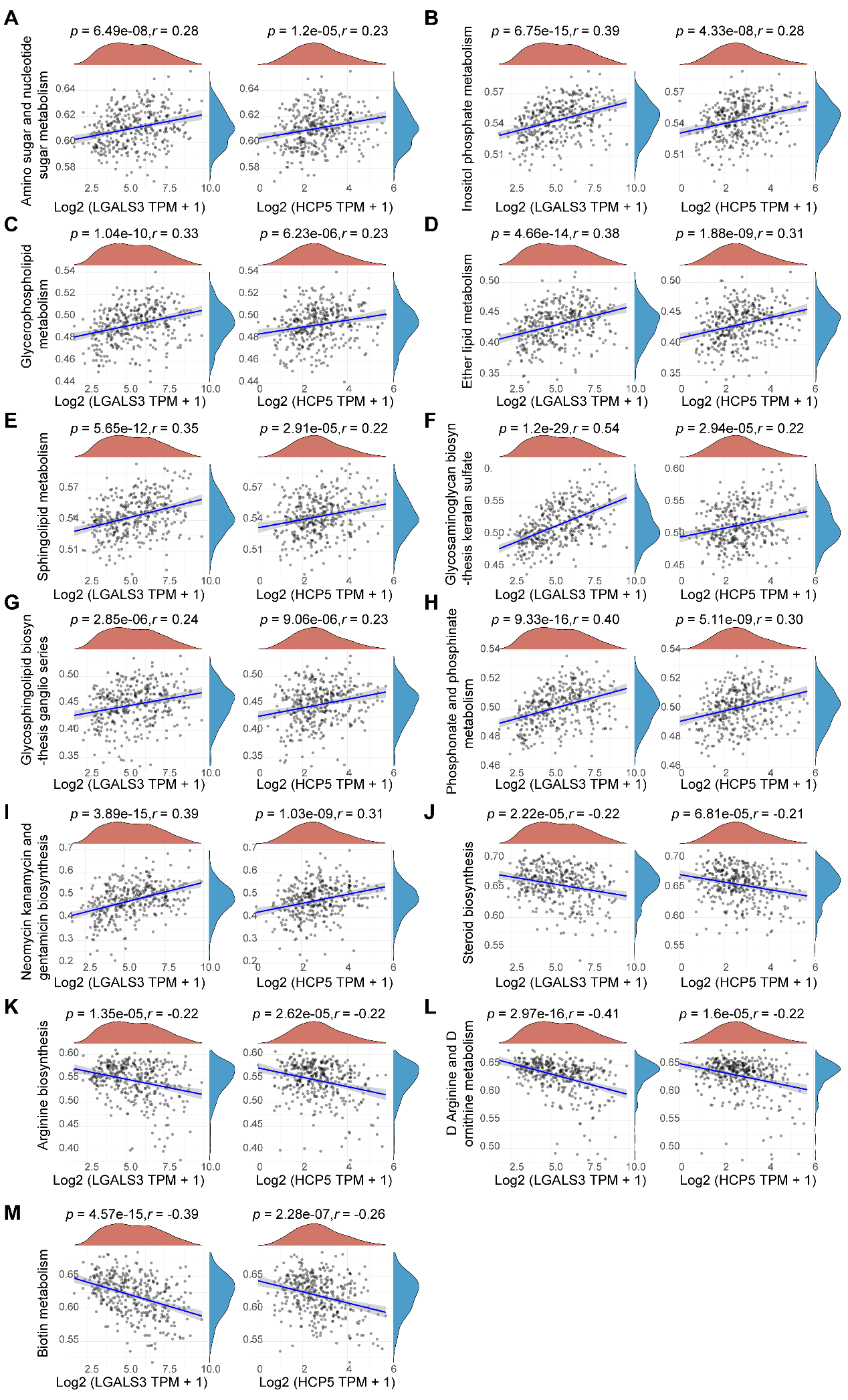


**Figure S5**. The correlation between LGALS3 or HCP5 gene and pathway score in HCC analyzed by Spearman method

The abscissa represents the expression of LGALS3 or HCP5 gene, and the ordinate represents the pathway score of this gene in HCC. The red curve represents the density distribution of LGALS3 or HCP5 gene, and the blue curve represents the distribution of pathway score. The positive correlation between LGALS3 or HCP5 and pathway score are given (|*r*| >0.2, *p* value < 0.05, n = 371).Thirteen metabolism-related pathways are included: (A) amino sugar and nucleotide sugar metabolism; (B) inositol phosphate metabolism; (C) glycerophospholipid metabolism, (D) ether lipid metabolism; (E) sphingolipid metabolism; (F) glycosaminoglycan biosynthesis keratan sulfate; (G) glycosphingolipid biosynthesis ganglio series; (H) phosphonate and phosphinate metabolism; (I) neomycin kanamycin and gentamicin biosynthesis; (J) steroid biosynthesis; (K) arginine biosynthesis; (L) D Arginine and D ornithine metabolism; (M) biotin metabolism.

**Table S1**. The correlation between LGALS3 expression and chemotactic activity of immune cells.

| Immune cell | Chemokine | *R* value | *p* value |
| --- | --- | --- | --- |
| Monocytes/macrophages | CCL2 | 0.43 | 1.2E-17*** |
|  | CCL3 | 0.19 | 0.00033*** |
|  | CCL5 | 0.3 | 2.3E-09*** |
|  | CCL7 | 0.24 | 3.9E-06*** |
|  | CCL8 | 0.081 | 0.11 |
|  | CCL13 | 0.24 | 3.4E-06*** |
|  | CCL17 | 0.23 | 8.6E-06*** |
|  | CCL22 | 0.37 | 3.6E-13*** |
| T lymphocytes | CCL2 | 0.43 | 1.2E-17*** |
|  | CCL1 | 0.14 | 0.0078** |
|  | CCL22 | 0.37 | 3.6E-17*** |
|  | CCL17 | 0.23 | 8.6E-06*** |
| Mast cells | CCR1 | 0.31 | 8.3E-10*** |
|  | CCR2 | 0.37 | 1.1E-13*** |
|  | CCR3 | 0.14 | 0.0064** |
|  | CCR4 | 0.34 | 1.6E-11*** |
|  | CCR5 | 0.42 | 8.4E-17*** |
|  | CXCR2 | 0.21 | 4.6E-05*** |
|  | CXCR4 | 0.46 | 4.3E-21*** |
| Eosinophils | CCL11 | 0.44 | 5.5E-19*** |
|  | CCL24 | 0.05 | 0.34 |
|  | CCL26 | 0.52 | 4.6E-27*** |
|  | CCL5 | 0.3 | 2.3E-09*** |
|  | CCL7 | 0.24 | 3.9E-06*** |
|  | CCL13 | 0.24 | 3.4E-06*** |
|  | CCL3 | 0.19 | 0.00033*** |
| Neutrophils | CXCL8 | 0.37 | 2.6E-13*** |

***p* value < 0.01; ****p* value < 0.001.

**Table S2**. Correlation analysis between LGALS3 and biomarkers of immune cells in HCC.

| Immune cell | Biomarker | *R* value | *p* value |
| --- | --- | --- | --- |
| B cell | CD19 | 0.26 | 2.7E-07*** |
|  | CD79A | 0.28 | 5.2E-08*** |
| CD8^+^ T cell | CD8A | 0.28 | 2.8E-08*** |
|  | CD8B | 0.24 | 4.4E-06*** |
| CD4^+^ T cell | CD4 | 0.3 | 5.5E-09*** |
| M1 macrophage | NOS2 | 0.14 | 0.0058** |
|  | IRF5 | 0.3 | 2.7E-09*** |
|  | PTGS2 | 0.41 | 5.1E-16*** |
| M2 macrophage | CD163 | 0.2 | 0.00013*** |
|  | VSIG4 | 0.33 | 1.4E-10*** |
|  | MS4A4A | 0.29 | 1.4E-08*** |
| Neutrophil | CEACAM8 | -0.026 | 0.62 |
|  | ITGAM | 0.35 | 7.3E-12*** |
|  | CCR7 | 0.28 | 2.6E-08*** |
| Dendritic cell | HLA-DPB1 | 0.38 | 8.1E-14*** |
|  | HLA-DQB1 | 0.26 | 5.5E-07*** |
|  | HLA-DRA | 0.36 | 7.3E-13*** |
|  | HLA-DPA1 | 0.36 | 7.9E-13*** |
|  | CD1C | 0.29 | 1.7E-08*** |
|  | NRP1 | 0.19 | 3E-04*** |
|  | ITGAX | 0.41 | 1.3E-16*** |
| CAFs | FAP | 0.54 | 2.2E-29*** |
|  | ACTA2 | 0.29 | 1.1E-08*** |
|  | S100A4 | 0.46 | 1.4E-20*** |
|  | PDPN | 0.48 | 5.7E-23*** |
|  | PDGFR | 0.38 | 5.9E-14*** |
|  | CD70 | 0.29 | 2.3E-08*** |

***p* value < 0.01; ****p* value < 0.001.

**Table S3**. Correlation analysis between HCP5 and biomarkers of immune cells in HCC.

| Immune cell | Biomarker | *R* value | *p* value |
| --- | --- | --- | --- |
| B cell | CD19 | 0.28 | 5.2E-08*** |
|  | CD79A | 0.29 | 1.3E-08*** |
| CD8+ T cell | CD8A | 0.42 | 1.9E-17*** |
|  | CD8B | 0.33 | 4.1E-11*** |
| CD4+ T cell | CD4 | 0.31 | 1.5E-09*** |
| M1 macrophage | NOS2 | 0.2 | 0.00012*** |
|  | IRF5 | 0.33 | 1.1E-10*** |
|  | PTGS2 | 0.32 | 5.8E-10*** |
| M2 macrophage | CD163 | 0.26 | 5.7E-07*** |
|  | VSIG4 | 0.37 | 3.6E-13*** |
|  | MS4A4A | 0.37 | 1.3E-13*** |
| Neutrophil | CEACAM8 | 0.054 | 0.3 |
|  | ITGAM | 0.44 | 7.2E-19*** |
|  | CCR7 | 0.38 | 2.3E-14*** |
| Dendritic cell | HLA-DPB1 | 0.46 | 2.7E-20*** |
|  | HLA-DQB1 | 0.27 | 9.3E-08*** |
|  | HLA-DRA | 0.53 | 9.5E-28*** |
|  | HLA-DPA1 | 0.48 | 6.7E-23*** |
|  | CD1C | 0.21 | 7.1E-05*** |
|  | NRP1 | 0.3 | 4.6E-09*** |
|  | ITGAX | 0.38 | 2.1E-14*** |
| CAFs | FAP | 0.26 | 3.6E-07*** |
|  | ACTA2 | 0.16 | 0.0018** |
|  | S100A4 | 0.29 | 9.3E-09*** |
|  | PDPN | 0.22 | 1.4E-05*** |
|  | PDGFR | 0.14 | 0.0065** |
|  | CD70 | 0.37 | 2.5E-13*** |

***p* value < 0.01; ****p* value < 0.001.

**Table S4**. The correlation between HCP5 expression and chemotactic activity for immune cells.

| Immune cell | Chemokine | *R* value | *p* value |
| --- | --- | --- | --- |
| Monocytes/macrophages | CCL2 | 0.36 | 4.6E-13*** |
|  | CCL3 | 0.22 | 1.7E-05*** |
|  | CCL5 | 0.43 | 5.7E-18*** |
|  | CCL7 | 0.23 | 1.2E-05*** |
|  | CCL8 | 0.35 | 4.6E-12*** |
|  | CCL13 | 0.28 | 5E-08*** |
|  | CCL17 | 0.18 | 0.00038*** |
|  | CCL22 | 0.32 | 4.4E-10*** |
| T lymphocytes | CCL2 | 0.36 | 4.6E-13*** |
|  | CCL1 | 0.17 | 0.0012** |
|  | CCL22 | 0.32 | 4.4E-10*** |
|  | CCL17 | 0.18 | 0.00038*** |
| Mast cells | CCR1 | 0.43 | 1E-17*** |
|  | CCR2 | 0.4 | 5.5E-16*** |
|  | CCR3 | 0.27 | 1.7E-07*** |
|  | CCR4 | 0.4 | 5.8E-16*** |
|  | CCR5 | 0.44 | 6.9E-19*** |
|  | CXCR2 | 0.32 | 1.6E-10*** |
|  | CXCR4 | 0.41 | 3.5E-16*** |
| Eosinophils | CCL11 | 0.25 | 1.9E-06*** |
|  | CCL24 | 0.13 | 0.011* |
|  | CCL26 | 0.24 | 2.6E-06*** |
|  | CCL5 | 0.43 | 5.7E-18*** |
|  | CCL7 | 0.23 | 1.2E-05*** |
|  | CCL13 | 0.28 | 5E-08*** |
|  | CCL3 | 0.22 | 1.7E-05*** |
| Neutrophils | CXCL8 | 0.27 | 1.5E-07*** |

**p* value < 0.05; ***p* value < 0.01; ****p* value < 0.001.

**Table S5.** The abbreviations and the analyzed tumors' full names throughout the present investigation.

| Abbreviations | Full names |
| --- | --- |
| CHOL | cholangiocarcinoma |
| ESCA | esophageal carcinoma |
| GBM | glioblastoma multiforme |
| KICH | kidney chromophobe |
| KIRC | kidney renal clear cell carcinoma |
| KIRP | kidney renal papillary cell carcinoma |
| LIHC | liver hepatocellular carcinoma |
| SKCM | skin cutaneous melanoma |
| THCA | thyroid carcinoma |
| UCEC | uterine corpus endometrial carcinoma |
| BLCA | bladder urothelial carcinoma |
| BRCA | breast invasive carcinoma |
| COAD | colon adenocarcinoma |
| HNSC | head and neck squamous cell carcinoma |
| LUAD | lung adenocarcinoma |
| LUSC | lung squamous cell carcinoma |
| PCPG | pheochromocytoma and paraganglioma |
| PRAD | prostate adenocarcinoma |
| READ | rectum adenocarcinoma |
| CESC | endocervical adenocarcinoma |
| PAAD | pancreatic adenocarcinoma |
| STAD | stomach adenocarcinoma |
| DLBC | lymphoid neoplasm diffuse large B-cell lymphoma |
| LAML | acute myeloid leukemia |
| THYM | thymoma |
| UCS | uterine carcinosarcoma |
| ACC | adrenocortical carcinoma |
| LGG | brain lower grade glioma |
| OV | ovarian serous cystadenocarcinoma |
| SARC | sarcoma |
| TGCT | testicular germ cell tumors |
| clear cell RCC | renal cell carcinoma |

**Table S6.** Patient characteristics.

| Number of patients | Sex | Age, years | Histology | Stage |
| --- | --- | --- | --- | --- |
| 1 | Male | 69 | HCC | Ⅰ |
| 2 | Male | 69 | HCC | Ⅰ |
| 3 | Female | 70 | HCC | Ⅰ |
| 4 | Male | 64 | HCC | Ⅰ |
| 5 | Female | 53 | HCC | Ⅰ |
